# Mapping of Multiple Complementary Sex Determination Loci in a Parasitoid Wasp

**DOI:** 10.1093/gbe/evz219

**Published:** 2019-10-09

**Authors:** Cyril Matthey-Doret, Casper J van der Kooi, Daniel L Jeffries, Jens Bast, Alice B Dennis, Christoph Vorburger, Tanja Schwander

**Affiliations:** 1 Department of Ecology and Evolution, University of Lausanne, Switzerland; 2 Groningen Institute for Evolutionary Life Sciences, University of Groningen, the Netherlands; 3 Institute of Integrative Biology, ETH Zürich, Switzerland; 4 Department of Aquatic Ecology, EAWAG, Swiss Federal Institute of Aquatic Science and Technology, Dübendorf, Switzerland; 5 Unit of Evolutionary Biology and Systematic Zoology, Institute of Biochemistry and Biology, University of Potsdam, Germany; 6 Department of Genomes and Genetics, Institut Pasteur, Paris, France

**Keywords:** hymenoptera, sex determination, *Lysiphlebus fabarum*, CSD

## Abstract

Sex determination has evolved in a variety of ways and can depend on environmental and genetic signals. A widespread form of genetic sex determination is haplodiploidy, where unfertilized, haploid eggs develop into males and fertilized diploid eggs into females. One of the molecular mechanisms underlying haplodiploidy in Hymenoptera, the large insect order comprising ants, bees, and wasps, is complementary sex determination (CSD). In species with CSD, heterozygosity at one or several loci induces female development. Here, we identify the genomic regions putatively underlying multilocus CSD in the parasitoid wasp *Lysiphlebus fabarum* using restriction-site associated DNA sequencing. By analyzing segregation patterns at polymorphic sites among 331 diploid males and females, we identify up to four CSD candidate regions, all on different chromosomes. None of the candidate regions feature evidence for homology with the *csd* gene from the honey bee, the only species in which CSD has been characterized, suggesting that CSD in *L. fabarum* is regulated via a novel molecular mechanism. Moreover, no homology is shared between the candidate loci, in contrast to the idea that multilocus CSD should emerge from duplications of an ancestral single-locus system. Taken together, our results suggest that the molecular mechanisms underlying CSD in Hymenoptera are not conserved between species, raising the question as to whether CSD may have evolved multiple times independently in the group.

## Introduction

A common mechanism of sex determination in animals is via genetic factors, for example, by sex chromosomes or sex-specific ploidy (Bachtrog et al. 2014). Haplodiploidy is a widespread genetic sex determination system found in ∼12% of all animal species (Bachtrog et al. 2014), encompassing some groups of beetles and mites, whiteflies, as well as the whole insect orders Thysanoptera (thrips) and Hymenoptera. In haplodiploid sex determination, unfertilized eggs develop into (haploid) males, and fertilized eggs develop into (diploid) females. In many haplodiploid hymenopteran species, the molecular mechanism underlying female development depends on heterozygosity at the complementary sex determination (CSD) locus (Crozier 1971; Heimpel and de Boer 2008). Female development is induced when the individual is heterozygous at the CSD locus, and male development is induced for individuals with only one allele at the CSD locus (either via homo- or hemizygosity). In the honey bee, the only organism where the CSD locus has been characterized so far, the gene complementary sex determiner (*csd*) is a paralog of *transformer* that emerged through gene duplication, a key gene in the sex determination pathway of insects (Hasselmann et al. 2008). The precise mechanism by which CSD regulates sex determination is still unknown, but it is believed that the formation of a heterodimer is key to triggering the female developmental pathway (Beye 2004).

CSD-based sex determination generates a significant genetic load under inbreeding, as low-allelic diversity results in the production of CSD-homozygous, diploid eggs. Depending on the species, the resulting diploid males can have reduced fertility and/or survival (Holloway et al. 1999). It is thought that multilocus CSD (ml-CSD), a derived mechanism, has been favored under these conditions (van Wilgenburg et al. 2006; Boer et al. 2008). In species with ml-CSD, female development is induced if at least one of the CSD loci is heterozygous. Thus, haploid eggs develop into males, as they are hemizygous for all loci, and diploid males are only produced if individuals are homozygous at all loci (Whiting and April 1943).

**Fig. 1.**
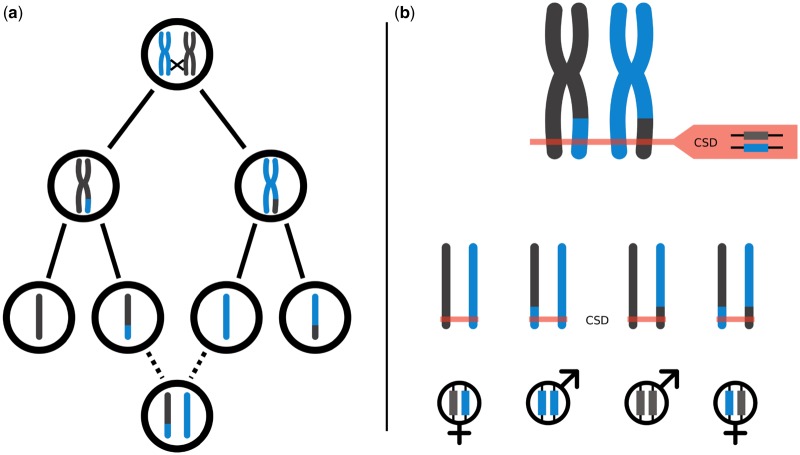
—Central-fusion automixis and CSD. (*a*) Parthenogenesis with central-fusion automixis and crossing over. Homologous chromosomes from the mother are represented in gray and blue, respectively. The oocyte undergoes normal meiosis, until two meiotic products originating from homologous chromosomes fuse to form a diploid egg. Chromosomal regions distal to a recombination event become homozygous. (*b*) Interaction between central-fusion and CSD. Visual representation of possible CSD genotypes in the case of a focal CSD locus distal to a recombination event. Assuming a recombination event occurred between the centromere and the CSD locus, an egg produced by central-fusion has a $\frac{1}{2}$ chance to develop into a diploid male. The proportion of recombinant offspring at random loci, should converge toward $\frac{2}{3}$ as the number of crossing over increases, thus, the chance for a heterozygous locus to become homozygous tends to $\frac{1}{3}$ as the number of recombinations increase (see Appendix A in Engelstädter et al. 2011).

CSD loci can be found by identifying genomic regions for which females are heterozygous and diploid males are always homozygous. In many species, diploid males are difficult to come by, because of their reduced fitness (Heimpel and de Boer 2008). However, we uncovered many diploid males while studying asexual reproduction (thelytokous parthenogenesis) in the parasitoid wasp *Lysiphlebus fabarum* (Hymenoptera: Braconidae), providing a rare opportunity to identify the CSD loci in this species. *Lysiphlebusfabarum* has both sexual and asexual lineages, and CSD is thought to consist of multiple loci, although the actual number of loci remains unknown (Engelstädter et al. 2011). In asexual *L. fabarum*, the cytological mechanism underlying thelytokous parthenogenesis is central-fusion automixis (Belshaw and Quicke 2003), which involves meiosis followed by a secondary restoration of diploidy through fusion of two meiotic products originating from homologous chromosomes. In this form of automixis, transitions to homozygosity and the associated production of diploid males happen in regions distal to recombination events (fig. 1*a*). Asexual production of females therefore predicts that at least some CSD loci are close to the centromeres, where heterozygosity is maintained in the long term (fig. 1*b*) (Vorburger 2014).

In this study, we explore the genetic basis of CSD in *L. fabarum* using 331 diploid males and females generated in a laboratory cross. Using restriction-associated DNA sequencing (RADseq) (Davey and Blaxter 2010) and association mapping, we identify regions that are highly homozygous in diploid males, and heterozygous in females. We identify four candidate CSD regions, all on different chromosomes and of which one is close to the putative centromeric region of its chromosome. These loci feature no homology to each other or the known CSD locus in the honey bee, suggesting that the molecular mechanism underlying haplodiploid sex determination is different in *L. fabarum* than in the species studied so far.

## Materials and Methods

All scripts and instructions required to reproduce the analysis are implemented in a pipeline available on Github at https://github.com/cmdoret/CSD_Lfabarum; last accessed October 16, 2019. For all analyses, we use contigs from the latest version of the *L. fabarum* reference genome (Dennis AB, in preparation) (Lfab v1.0, Available on request: https://bipaa.genouest.org/is/parwaspdb; last accessed October 16, 2019) which have been anchored onto six chromosomes (supplementary table S2, Supplementary Material online) in line with the six chromosomes that were deduced from karyotyping (Belshaw and Quicke 2003). Raw reads are available on NCBI SRA database under bioproject PRJNA505237, while the anchored genome used here along with all files required to reproduce the analysis are available on Zenodo at doi: 10.5281/zenodo.1488602.

### Samples and RADseq Protocol

Samples were obtained from a breeding experiment that was originally designed to introgress asexuality-causing allele(s) into a sexual line, with the aim to study the genetic basis of asexuality (supplementary fig. S1, Supplementary Material online). A haploid male coming from an asexual line of *L. fabarum* was crossed with two females from an iso-female sexual line. Following a crossing design similar to that used in Sandrock and Vorburger (2011), asexual females were obtained in the F3 generation (supplementary fig. S1, Supplementary Material online). These asexual females produced diploid sons and daughters, which are the focus of the present study. In total, we used 569 individuals from 45 families, including 11 F3 mothers, 153 F4 daughters, and 405 F4 sons. The samples were kept in ethanol at −20 °C until DNA extraction using a Quiagen DNeasy Blood & Tissue Kit, following manufacturer instructions. Individuals were sequenced in six separate libraries, following the protocol from Parchman et al. (2012), with the enzymes EcoRI and MseI, and size selection on agarose gel (200–450 bp). Samples were multiplexed in each library following the TruSeq multiplexing design, and libraries were pooled by pairs on the same Illumina lane using adapters iA06 or iA12. Single-end sequencing was performed using Illumina Hiseq 2500.

### STACKS Pipeline

We used the STACKS software (version 1.48) (Catchen et al. 2013) to process RADseq data. Following quality control using fastqc (https://www.bioinformatics.babraham.ac.uk/projects/fastqc; last accessed October 16, 2019, version 0.11), the raw reads were trimmed and demultiplexed using the “process radtags” module from the STACKS suite and two mismatches were allowed to detect adapters. The 93-bp trimmed, demultiplexed reads were aligned to the latest assembly of the *L. fabarum* genome using BWA-aln (version 0.7.2) (Li and Durbin 2009) with four mismatches allowed. Only uniquely aligned reads were extracted using samtools (version 1.4) (Li et al. 2009). Stacks were then generated from SAM files of unique hits using the Pstacks module, requiring a minimum read depth (-m) of 3 to consider a stack. Individuals with <10% uniquely aligned reads compared with the average of all samples were excluded from the analysis. The catalog of loci was built with Cstacks allowing for a distance (-n) of three mismatches between samples at each locus. The stacks populations module was run on all samples together, requiring each locus to be present (-r) in at least 80% of samples and have at least a sequencing depth (-m) of 20 for ploidy separation, or 5 for association mapping (supplementary table S1, Supplementary Material online). Using a higher coverage threshold for the association mapping results in fewer loci that can be analyzed, but does not change the main conclusions. We also required a minimum allele frequency (–min-maf) of 10%. The different STACKS parameters were selected following guidelines in Paris et al. (2016).

### Ploidy Separation and Filtering

To determine the ploidy of all individuals, we rely on genome-wide homozygosity. Haploid samples are hemizygous and should have extremely high homozygosity levels compared with diploid ones. We included only high-confidence SNPs in the STACKS populations module by using a stringent cutoff of 20 reads for the minimum sequencing depth (-m parameter). 899 high-confidence SNPs passed the quality filters and on an average, each sample presented 853 of these polymorphic sites with a (high) mean coverage of 132×. We then computed the proportion of homozygous SNPs per individual using VCFtools (version 0.1.13) (Danecek et al. 2011) on the output VCF file from populations. To account for sequencing errors and paralog merging, a conservative threshold of 90% homozygosity among polymorphic sites was determined empirically based on the bimodal distribution of homozygosity (supplementary fig. S2, Supplementary Material online). Individuals above that threshold (n = 196) were considered haploid. All these haploid individuals were males.

Haploid males were used to identify and filter out heterozygous SNPs generated via paralog merging. To this end, we extracted loci that were heterozygous in >50% of haploid samples (26 loci) and removed these from the set of loci to analyze in diploid samples. This was done by rerunning populations only on diploids and specifying the list of loci with heterozygous sites in haploids using the blacklist (-B) parameter.

### Association Mapping

Case–control association mapping was used to identify CSD-candidate regions, based on the observed heterozygosity at each SNP in males and females. The number of heterozygous males, heterozygous females, homozygous males, and homozygous females was computed for every SNP and a one-sided Fisher’s exact test was performed for each SNP on the 2×2 contingency table. The alternative hypothesis was that the proportion of homozygous males at the SNP is higher than the proportion of homozygous females. *P* values were corrected for multiple testing using Benjamini–Hochberg correction.

### Centromere Identification

The proportion of recombinant offspring per locus along the genome was used to estimate centromere position. In each family, all sites that are heterozygous in the mother were used. If the mother was not available, a site was considered heterozygous if at least one of her offspring was heterozygous or if two offspring were homozygous for different alleles. At each site, the proportion of recombinant (homozygous) offspring was computed among all offspring whose mother was heterozygous (all families pooled). The proportions were then used to visualize recombination rates along the genome using two different methods: 1) computing mean homozygosity in a sliding window containing 30 sites with a step size of 1, and 2) using a weighted local regression model of degree 2 with a span of 0.4 to obtain a smooth estimate curve. The weights given to each site in the local regression correspond to the number of offspring taken into account when computing the proportion of homozygous offspring. For each chromosome, the minimum value of the local regression curve was used to approximate centromere location.

### Nucleotide Diversity

Whole-genome sequencing was performed on 15 *L. fabarum* wild samples (11 haploid males and 4 females from the same population in a single geographic region) using paired-end reads on Illumina HiSeq 3000. The raw reads were trimmed using trimmomatic with LEADING: 20 and TRAILING: 20 and aligned to the reference genome using BWA-mem with default parameters. SNPs were then called using samtools mpileup (version 1.4) (Li et al. 2009) skipping indels, and variants aligning to chromosome-anchored contigs were extracted. *π* nucleotide diversity was computed in sliding windows of 100 bp with a step size of 10 bp.

### Recombination Rates

Recombination rates along the genome were interpolated from the same linkage map that was used to anchor the assembly. Recombination rates are assumed to be uniform between linkage map markers. Thus, the genetic distance between a linkage map marker and a SNP is linearly dependent on their physical distance. Given a SNP S between two linkage map markers M0 and M1 the genetic distance between S and M0 $G_{M0-S}$ is given by $G_{M0-S}=G_{M0-M1}*\frac{P_{M0-S}}{P_{M0-M1}}$ where *P* are physical distances in base pairs.

### Collinearity Analyses

Collinearity blocks were defined using the default parameters of MCScanX: A collinearity block is called if two genomic segments share 5 homologous genes in conserved order with at most other 25 genes inserted in between. Gene coordinates were defined by merging maker gene prediction tracks and transcripts assembled from reference-aligned RNAseq reads from 5-day-old larvae (Dennis AB, Käch H, Vorburger C, in revision) (SRA accession numbers SAMN10024115–SAMN10024165). Gene sequences were extracted at the merged coordinates using bedtools2 (Quinlan and Hall 2010) and homologous genes were defined by all versus all BlastN, using the BLAST+ command line tools (Camacho et al. 2009), selecting matches with an e-value inferior to 10e-05.

### Transformer Homology

Protein sequences of *feminizer*/*transformer* homologs were retrieved from UniproKB for eight species of Hymenoptera: *Nasonia vitripennis* (B3VN92), *Euglossa hemichlora* (D9MZ89), *Bombus terrestris* (B4Y115), *Melipona compressipes* (B4XU23), *Apis florea* (A0A0H4URN0), *Apis cerana* (B1NW84), *Apis mellifera* (Q6V5L4), and *Apis dorsata* (B1NW85). Homology with these sequences was searched using TBlastN against the *L. fabarum* genome.

## Results

### Samples and Sequencing

Our crossing design (Methods and supplementary fig. S1, Supplementary Material online) generated 45 families consisting of virgin asexual mothers with (diploid) daughters and both haploid and diploid sons. We genotyped 569 individuals of the 45 families by RADseq, via aligning to an available *L. fabarum* reference genome (see methods for details). After excluding 42 individuals with poor alignment statistics (<10% aligned reads compared with the average of all samples), we used STACKS (version 1.48) to generate a SNP catalog from the 527 remaining samples (380 males, 147 females; see methods for details).

In addition to diploid males and females, virgin asexual females produce haploid males. Such vestigial (haploid) male production is fairly common in asexuals (Sandrock and Vorburger 2011; van der Kooi and Schwander 2014). Since our approach relies on the comparison of heterozygosity between diploid males and females, haploid males are not informative. Because haploid and diploid males are phenotypically identical in *L. fabarum*, we used 899 high-confidence SNPs with a minimum sequencing depth of 20 to distinguish them. We considered all males with >90% monoallelic polymorphic sites as haploids (supplementary fig. S2, Supplementary Material online). Using this strategy, we removed 196 haploid males from the data set and kept 184 diploid males for further analyses.

### Identification of CSD Regions

**Fig. 2.**
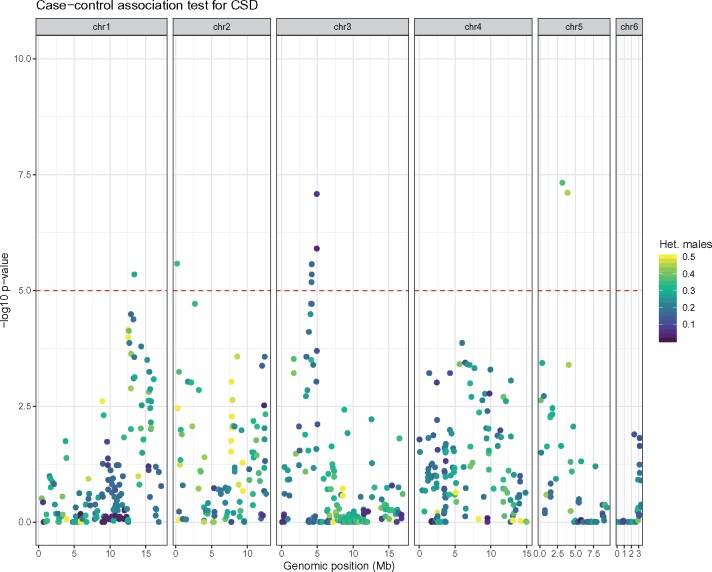
—Four CSD candidate regions in *Lysiphlebus fabarum*. Association mapping using one-sided Fisher’s exact tests for identifying SNPs with an excess of heterozygotes in females relative to males. The Manhattan plot shows −log10 *P* values, after Benjamini–Hochberg correction for multiple testing. The different panels show data for different chromosomes, with the horizontal red dashed line showing the *P* = 10e-5 threshold. SNPs are colored according to the proportion of heterozygous males.

After excluding haploid males, SNP calling was performed again on the 331 diploid individuals (males and females) with a more permissive sequencing depth filter (Methods, STACKS pipeline; Catchen et al. 2013), yielding 1,195 SNPs (corresponding to an expected density of one SNP every 65 kb on the 140-Mb *L. fabarum* genome). On an average, each sample presented 1,143 SNPs with a mean coverage of 100×. Of the 1,195 SNPs, 723 (61%) were on contigs anchored on chromosomes and were used in subsequent analyses. To identify CSD candidate regions, we then used a case–control design where we test the association of allelic state (homozygous and heterozygous) with sex (male and female). For each SNP, we performed a Fisher’s exact test to calculate a score based on the relative proportion of homozygous males versus females. High-scoring SNPs are therefore consistently found in a heterozygous state in females and/or in a homozygous state in males. After multiple testing correction (FDR = 10^−5^), we found four candidate regions, all on different chromosomes (fig. 2), of which two, on chromosomes 3 and 5, are highly significant (*P* < 10^−6^). Estimated recombination rates between separate SNPs in each region were homogeneous, suggesting the presence of a single CSD locus per region (supplementary fig. S3, Supplementary Material online).

We further assessed the fit of each candidate region to expected heterozygosity levels of CSD regions. According to the CSD model, diploid males must be homozygous at all CSD loci, whereas females need only be heterozygous at one locus. Candidate regions on chromosomes 1, 2, and 3 show a low proportion of heterozygous males (10–30%), while this proportion is much higher on the region from chromosome 5 (40–50%, fig. 2), meaning that the support for the CSD candidates on chromosomes 1, 2, and 3 is stronger than for candidates on chromosome 5.

We also attempted to use polymorphism levels, which are expected to be high for CSD loci, as an additional approach to compare CSD candidate regions. Diploid males having reduced fitness, rare CSD alleles are under positive selection in the wild, leading to balancing selection at CSD loci (Gloag et al. 2016). Regions undergoing such balancing selection could show elevated levels of nucleotide diversity in wild populations. This was shown to be the case for the CSD locus of the honey bee (Hasselmann and Beye 2004). We quantified diversity levels across the genome in *L. fabarum* using whole-genome sequencing data from 15 individuals randomly collected in a single natural population, but we did not detect a significant rise in diversity around any of the candidate regions (mean: 0.0041, SD: 0.0042) compared with the rest of the genome (mean: 0.0029, SD: 0.0055) (supplementary fig. S4, Supplementary Material online). This might be explained by much weaker balancing selection on each individual CSD locus under ml-CSD as in *L. fabarum* than on the one locus in sl-CSD species such as the honey bee. Similarly low diversity at the CSD locus was recently reported in the dwarf honeybee *Apis florea*, where it was proposed that a recent population bottleneck may have reduced the effect of balancing selection (Biewer et al. 2016).

### Location of Centromeric Regions

As CSD regions are expected to be close to centromeres in asexual *L. fabarum* (Engelstädter et al. 2011), we identified the most likely location of the centromere on each chromosome of the *L. fabarum* genome. Under central-fusion automixis, the genotype of any diploid offspring should be identical to that of their mother, except for regions distal to recombination events that can become homozygous (fig. 1); this causes homozygosity to increase with distance from the centromeres. Thus, for a locus that is heterozygous in the mother, the proportion of homozygous offspring (male or female) can be used as a proxy for the distance of that locus to the centromere.

**Fig. 3.**
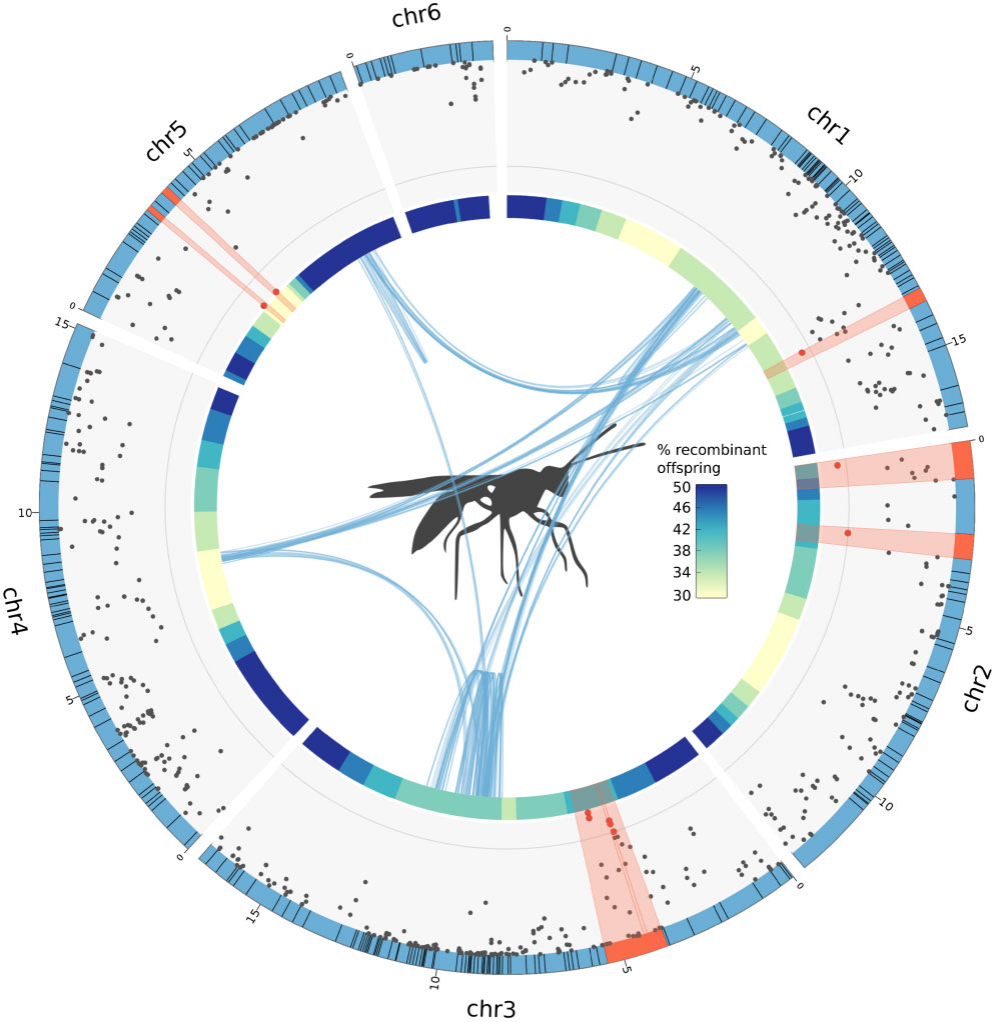
—Position of CSD candidate regions relative to centromeres and collinearity blocks. This plot integrates three layers of information from the current study. Each blue segment forming the outer circle represents a chromosome and the black lines intersecting them illustrate the boundaries of anchored contigs. The scatterplot shows the Manhattan plot from figure 2 with highly significant (*P* < 10^−5^, gray line) SNPs and their corresponding contigs shown in red. The inner colored circle is a heatmap showing the likely location of the centromeres along chromosomes, estimated from the proportion of recombinant (homozygous) offspring at mother-heterozygous sites, from low (yellow) to high (blue) (supplementary fig. S5, Supplementary Material online). Blue curves in the middle show collinearity blocks obtained using MCScanX with default parameters.

Using this approach, we could infer the likely location of the centromeric regions for five out of six chromosomes (supplementary fig. S5, Supplementary Material online). Modeling recombination rates using both weighted local regression and moving averages yielded similar results (supplementary fig. S5, Supplementary Material online). For the sixth chromosome, which has a smaller number of markers, it was impossible to make a reliable inference of the centromeric region. Out of the four candidate CSD regions, only the candidate on chromosome 5 is close to the estimated centromere location (2 Mb). Other candidates on chromosomes 1, 3, and 5 are not significantly closer to centromeres than the rest of polymorphic sites (fig. 3 andsupplementary fig. S7, Supplementary Material online). Close proximity would be expected in organisms with central-fusion automixis, as CSD loci that are further from centromeres would be rendered homozygous in case of recombination (Vorburger 2014), causing the loss of their heterozygosity-dependent feminizing effect.

### Collinearity across CSD Regions

The molecular mechanisms underlying ml-CSD have not been studied thus far, but a verbal model suggested that ml-CSD may derive from a sl-CSD system via duplication of the original CSD locus (Boer et al. 2008; Schmieder et al. 2012). We therefore evaluated whether the multiple CSD regions of *L. fabarum* could have evolved via duplication. A common approach to infer gene duplications across different genomic regions is to look for collinearity; the conserved order of homologous genes between regions. We used MCScanX (Wang et al. 2012) to investigate genome-wide patterns of collinearity in genes and transcripts. The coordinates used were generated by combining gene tracks from the MAKER annotation pipeline (Dennis AB, in preparation) and coordinates from aligned transcripts from larvae (Dennis AB, Käch H, Vorburger C, in revision) (SRA accession numbers SAMN10024115–SAMN10024165). We found no evidence for collinearity between candidate CSD regions, suggesting the different CSD loci in *L. fabarum* did not evolve via duplication (fig. 3). A genuine absence of collinearity could mean either that the genetic elements differ across loci, or that the similar region is not large enough to be detected using collinearity. It is also possible that the assembly is too fragmented to detect collinearity, with unanchored contigs interrupting collinearity blocks inside chromosomes. Indeed, the genome is split into 1,698 contigs of which 296, accounting for 53.5% of the assembly length, were anchored to chromosomes using a linkage map (supplementary table S2, Supplementary Material online). However, this should not prevent us from identifying a paralogy across CSD regions, as the association mapping revealed very few high-scoring SNPs in unanchored contigs (supplementary fig. S6, Supplementary Material online).

Improving the placement of contigs or the genome assembly in future studies will allow to reduce the technical constraints for detecting paralogy. Nonetheless, the association mapping step is not affected by genome completeness and identified CSD candidate regions lay the foundations for more detailed molecular characterization of each region.

### Transformer Homology

The upstream molecular mechanisms underlying CSD in *L. fabarum* are likely different from those in the honey bee. Following its duplication from *transformer* (called *feminizer* in the honeybee), the honey bee *csd* gene has been under strong positive selection, resulting in its neofunctionalization as the master switch for sex determination. To investigate whether a homolog of the *transformer* gene is present in the *L. fabarum* candidate CSD regions, we retrieved the protein sequences of *transformer* homologs from eight different hymenopteran species (Methods, Transformer homology) and searched the *L. fabarum* genome using TBlastN. This approach allowed us to identify a *transformer* homolog on chromosome 1 at 7 Mb (positions: 7,006,657–7,006,839), but there was no homolog in any of the candidate CSD regions. There was also no additional *transformer* homolog in the unanchored contigs. The absence of *transformer* homologs in the *L. fabarum* CSD regions suggests that the CSD in *L. fabarum* is based on different molecular mechanisms than in the honey bee. As we were able to identify a *transformer* homolog elsewhere in the genome, our results are unlikely due to the *L. fabarum transformer* sequence being too diverged for identification via homology searches (Mine et al. 2017).

## Discussion

We studied the CSD system in the parasitoid wasp *L. fabarum* and identified up to four candidate CSD regions on different chromosomes. The absence of a transformer homolog in any of these regions suggests a novel molecular mechanism underlying CSD in *L. fabarum*, with an upstream cue that differs from the one in the honey bee. The other nonhoney bee species with genomic candidate regions for CSD, the ant *Vollenhovia emeryi*, possesses two *transformer* copies in one of the candidate regions, which led to the suggestion of a conserved CSD mechanism across ants and bees (Miyakawa and Mikheyev 2015). Based on our results, such conservation does not seem extend to braconid wasps, a clade that diverged from ants and bees ∼200 Ma (Peters et al. 2017).

Our findings suggest that CSD in *L. fabarum* is based on up to four separate loci, in line with previous inferences based on high variation of diploid male production among different lines of asexual females (Engelstädter et al. 2011). However, the exact number of different CSD loci in *L. fabarum* remains unknown. For example, there could be additional polymorphic CSD loci in wild populations that were fixed in our studied crosses. Furthermore, the level of support varies among the four loci identified in our study. The candidate locus on chromosome 3 is supported by the highest number of SNPs and shows the most significant association between heterozygosity and sex (*P* < 10^−7^). By contrast, the candidate region on chromosome 5 is highly heterozygous in females, but a high proportion of males are also heterozygous, making it a less promising candidate, as males should be homozygous at all CSD loci. This genetic region could, for example, contain genetic factors unrelated to CSD, but be potentially lethal to females when present in a homozygous state, while having no particular effect on males.

Ml-CSD should be favored in species with asexual reproduction via automixis (as in *L. fabarum*) or high inbreeding, where it would decrease the load caused by diploid male production (Vorburger 2014). Our laboratory cross was designed to generate new asexual strains via introgression of asexuality-causing alleles into the genetic background of a sexual species. As a consequence, centromere regions that would never become homozygous under asexuality were rendered homozygous via inbreeding in the sexual generations, resulting in the frequent production of diploid males by the new asexual strains. In wild asexual populations however, loci close to the centromeres will remain heterozygous because of central-fusion automixis. CSD loci in asexual populations should therefore be preferentially located in centromeric regions to minimize the production of diploid males. Surprisingly, we only found one out of four putative CSD loci to be particularly close to centromeric regions (fig. 3). This means that positive selection of heterozygous genotypes rather than proximity to centromeres may drive the maintenance of heterozygosity at CSD regions, as was shown in the cape honeybee (Smith et al. 2019).

Our results call for a reconsideration of the existing theoretical model for the evolution and functioning of ml-CSD. Multilocus CSD is thought to originate by duplication of an ancestral, single CSD locus (van Wilgenburg et al. 2006; Boer et al. 2008). However, the duplication model raises several questions. For example, it does not explain why two recently duplicated CSD loci hemi- or homozygous for different alleles would not be able to complement each other and generate haploid females. Nonetheless such individuals are unheard of in CSD species. In light of our results, it seems more likely that the different CSD loci have different functions and were not generated via duplication but recruited independently as upstream signals in sex determination. Perhaps the amount of signal generated could differ among loci and the threshold required to trigger female development could be reached with only a subset of heterozygous loci. This would explain the different strengths of association for the different candidate loci. There are currently two known genetic mechanisms underlying haplodiploid sex determination in Hymenoptera (Van De Zande and Verhulst 2014): sl-CSD, in the honey bee *Apis mellifera*, and parental genome imprinting, in the jewel wasp *Nasonia vitripennis* (Beukeboom and van de Zande 2010). Functional investigation of the CSD regions in *L. fabarum* may reveal a third molecular mechanism of sex determination in Hymenoptera. 

## Supplementary Material

Supplementary data are available at *Genome Biology and Evolution* online.

## Supplementary Material

evz219_Supplementary_Data

## Notes

**Data deposition:** This project has been deposited at the NCBI BioProject database under accession PRJNA505237.
