# Supplementary figures and images for: Mapping of Multiple Complementary Sex Determination Loci in a Parasitoid Wasp
Source: Genome Biol Evol. 2019 Oct 9;11(10):2954–62. doi: 10.1093/gbe/evz219 (PMC6821247; doi:10.1093/gbe/evz219)

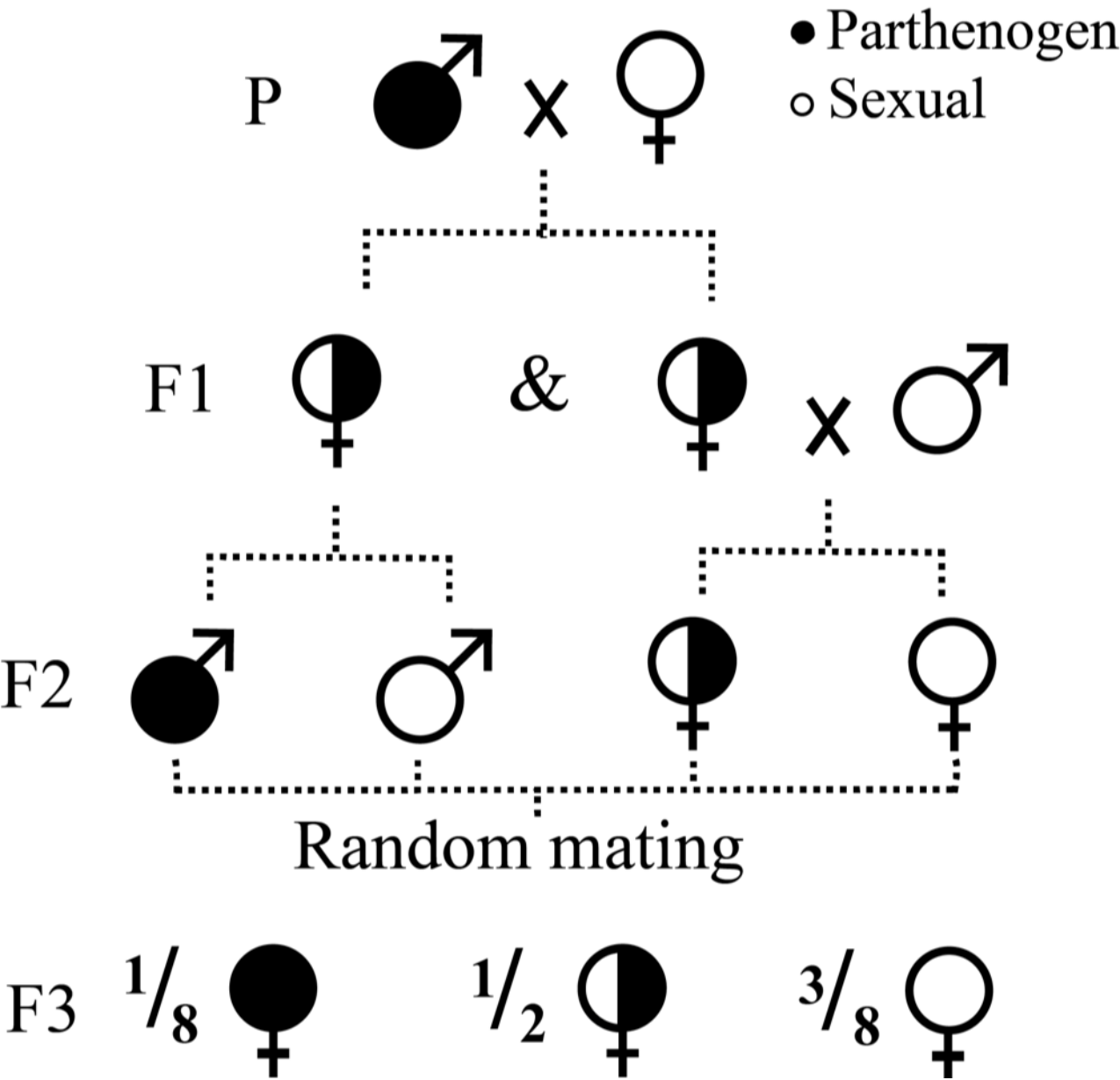

Supplement: evz219_Supplementary_Data [file evz219_supplementary_data.zip › fig_S1.pdf]

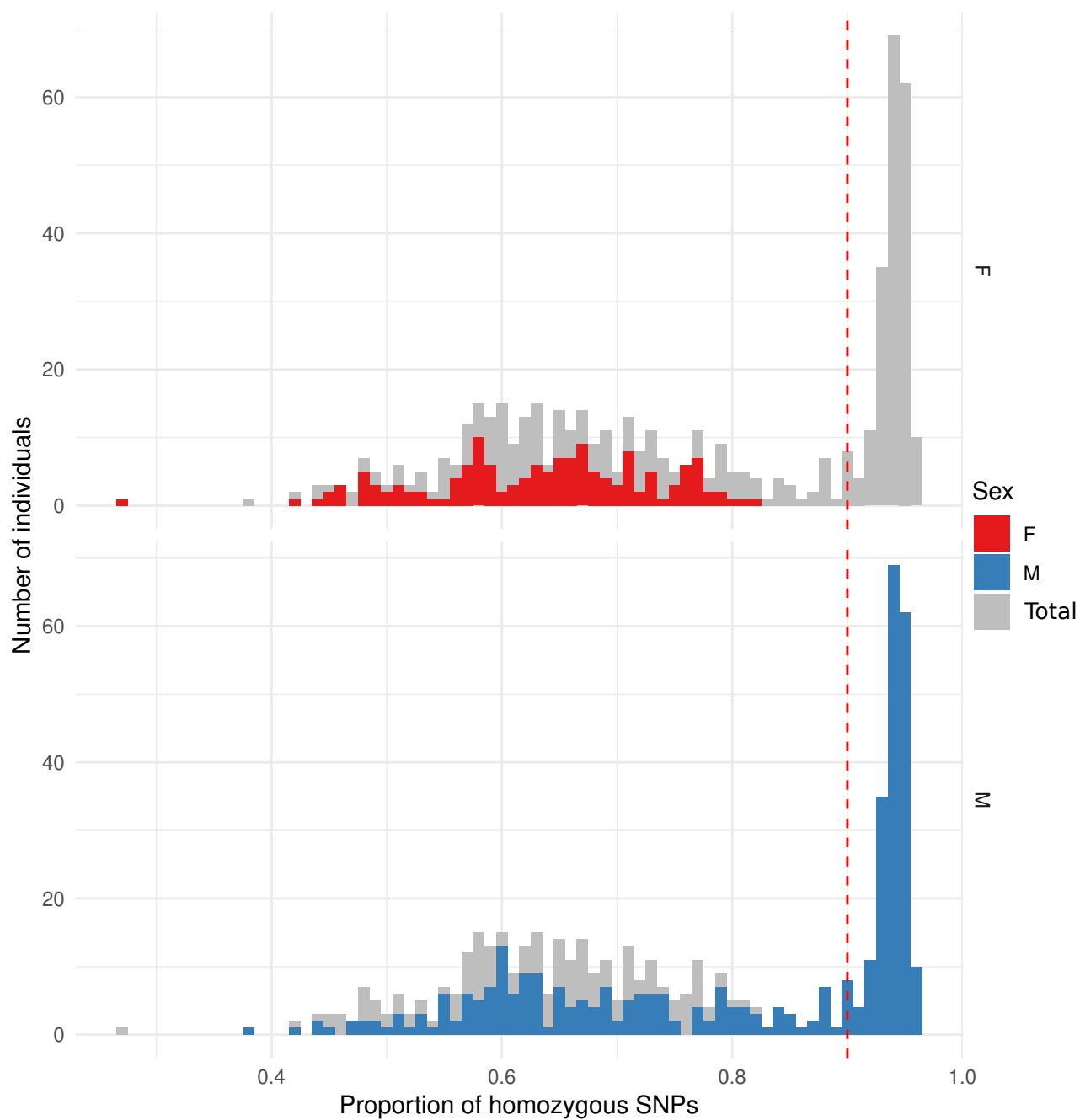

Supplement: evz219_Supplementary_Data [file evz219_supplementary_data.zip › fig_S2.pdf]

# Case-control association test for CSD

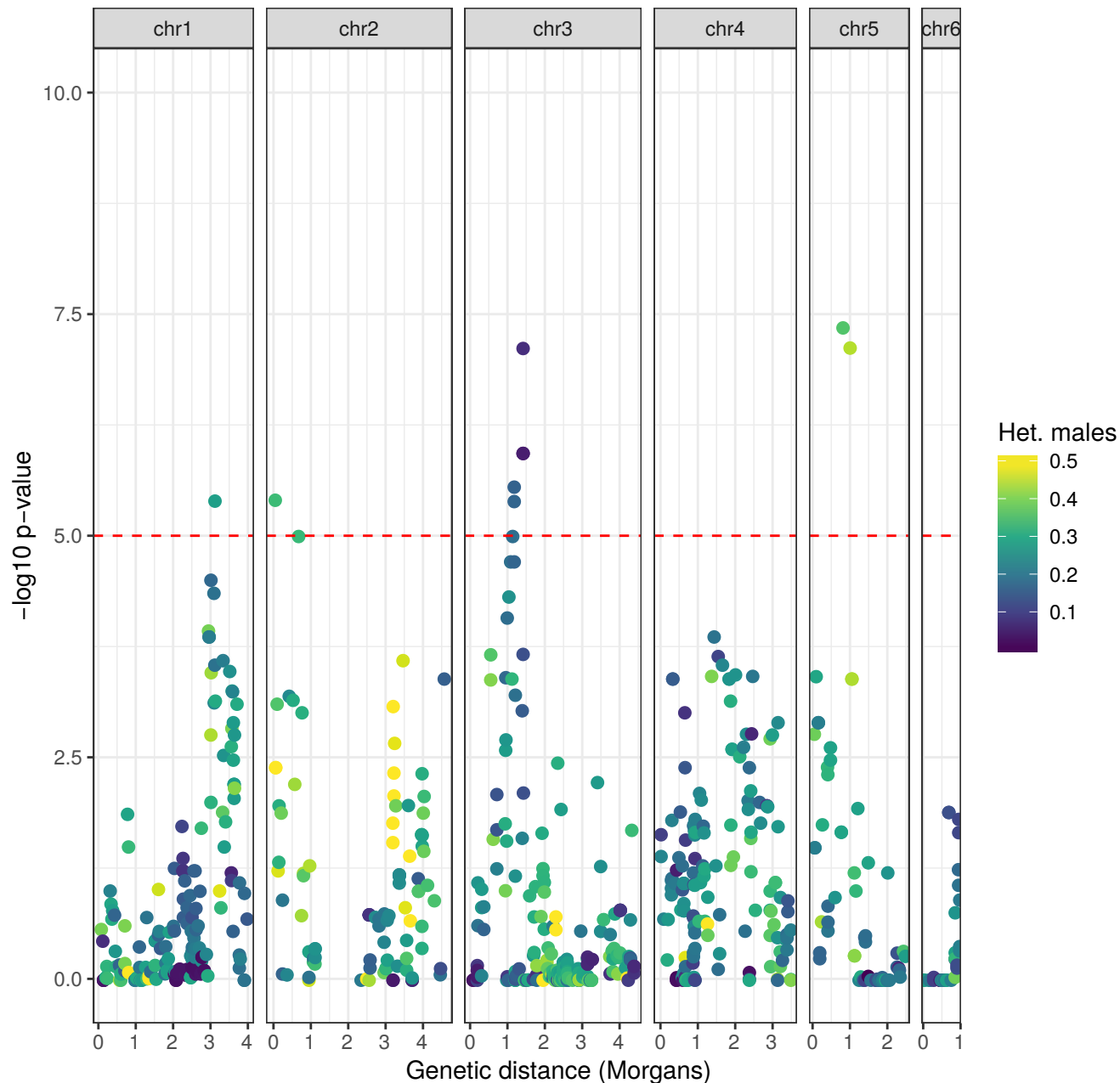

Supplement: evz219_Supplementary_Data [file evz219_supplementary_data.zip › fig_S3.pdf]

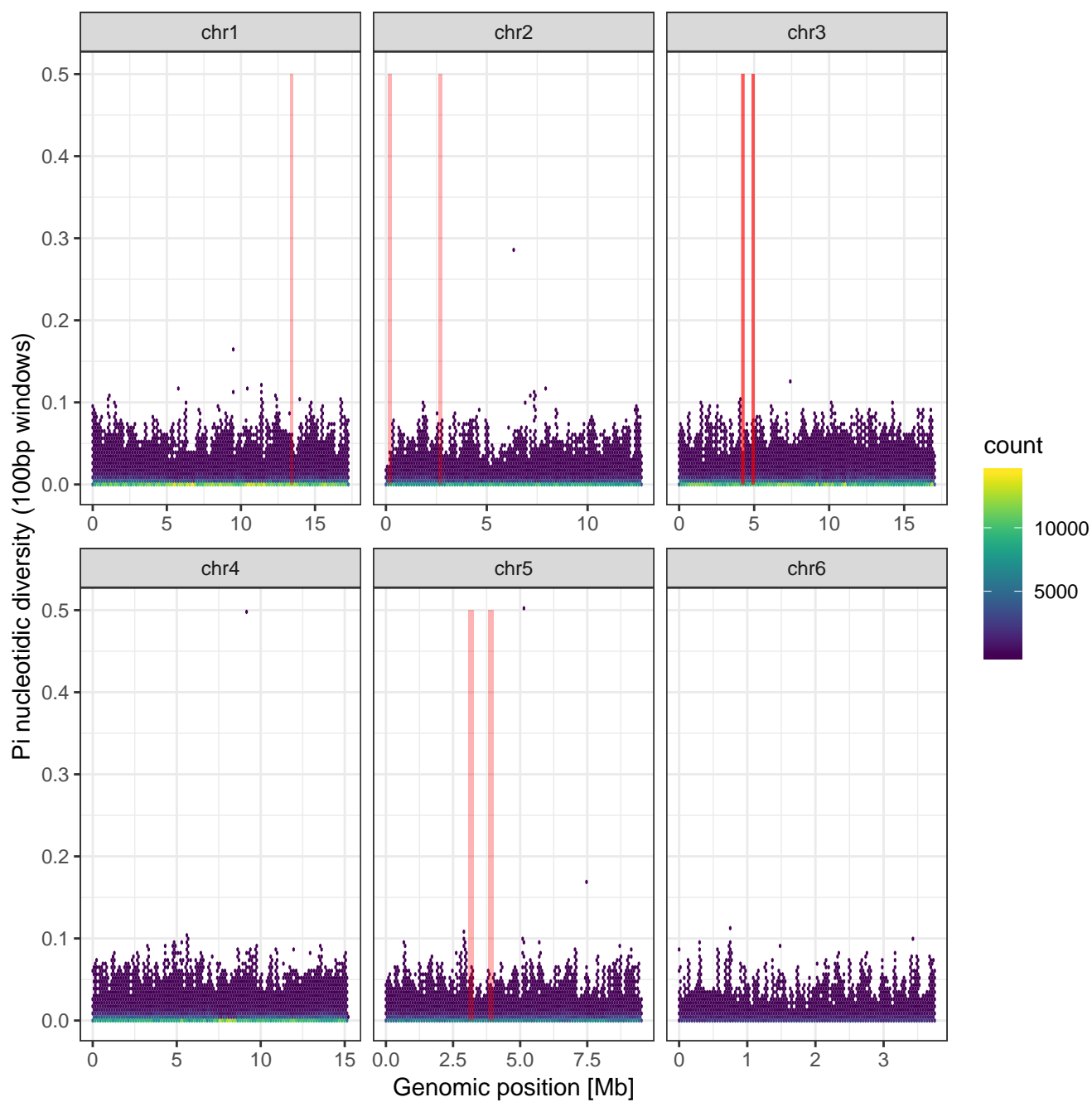

Supplement: evz219_Supplementary_Data [file evz219_supplementary_data.zip › fig_S4.pdf]

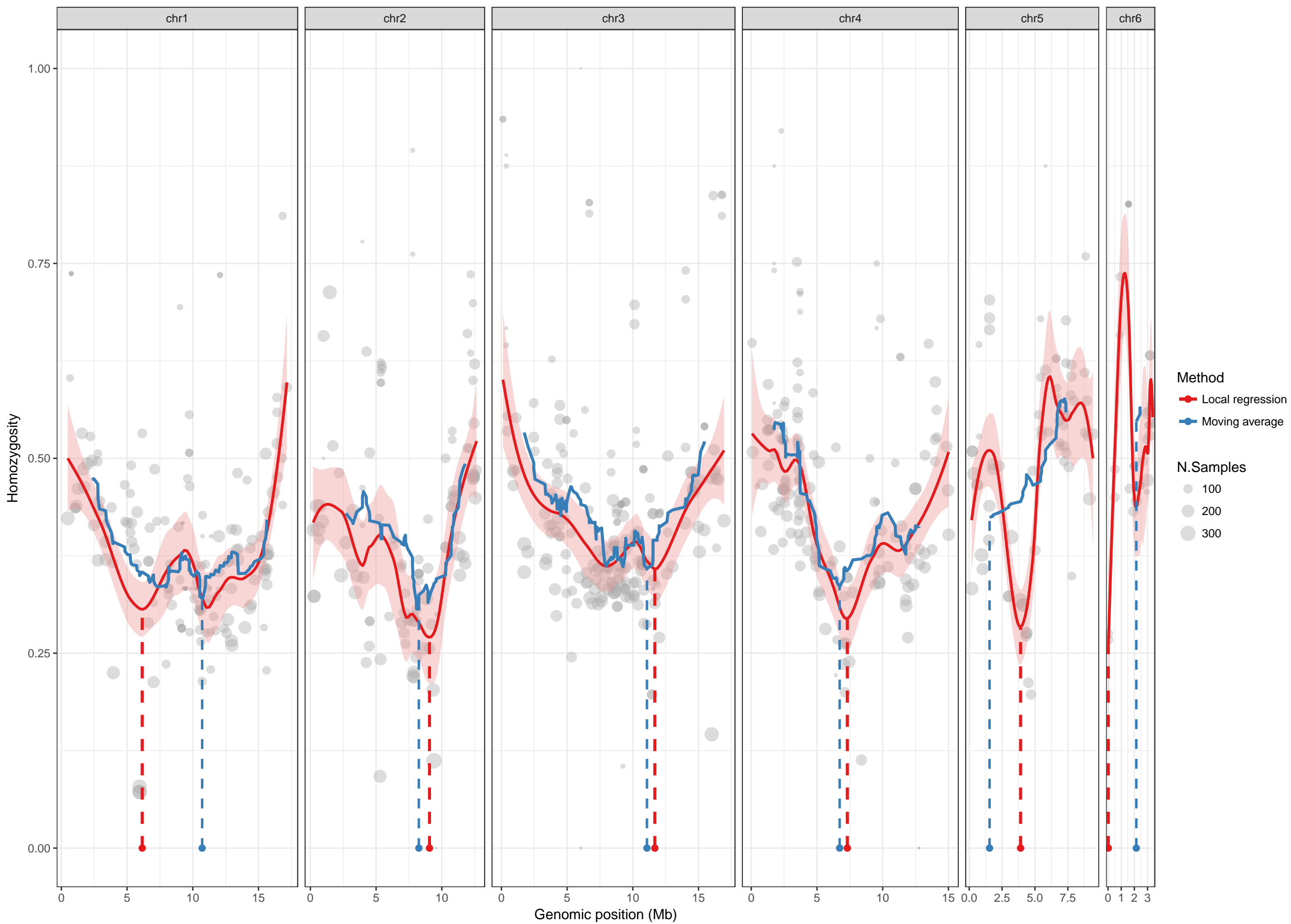

Supplement: evz219_Supplementary_Data [file evz219_supplementary_data.zip › fig_S5.pdf]

Case-control association test for CSD: Unordered contigs

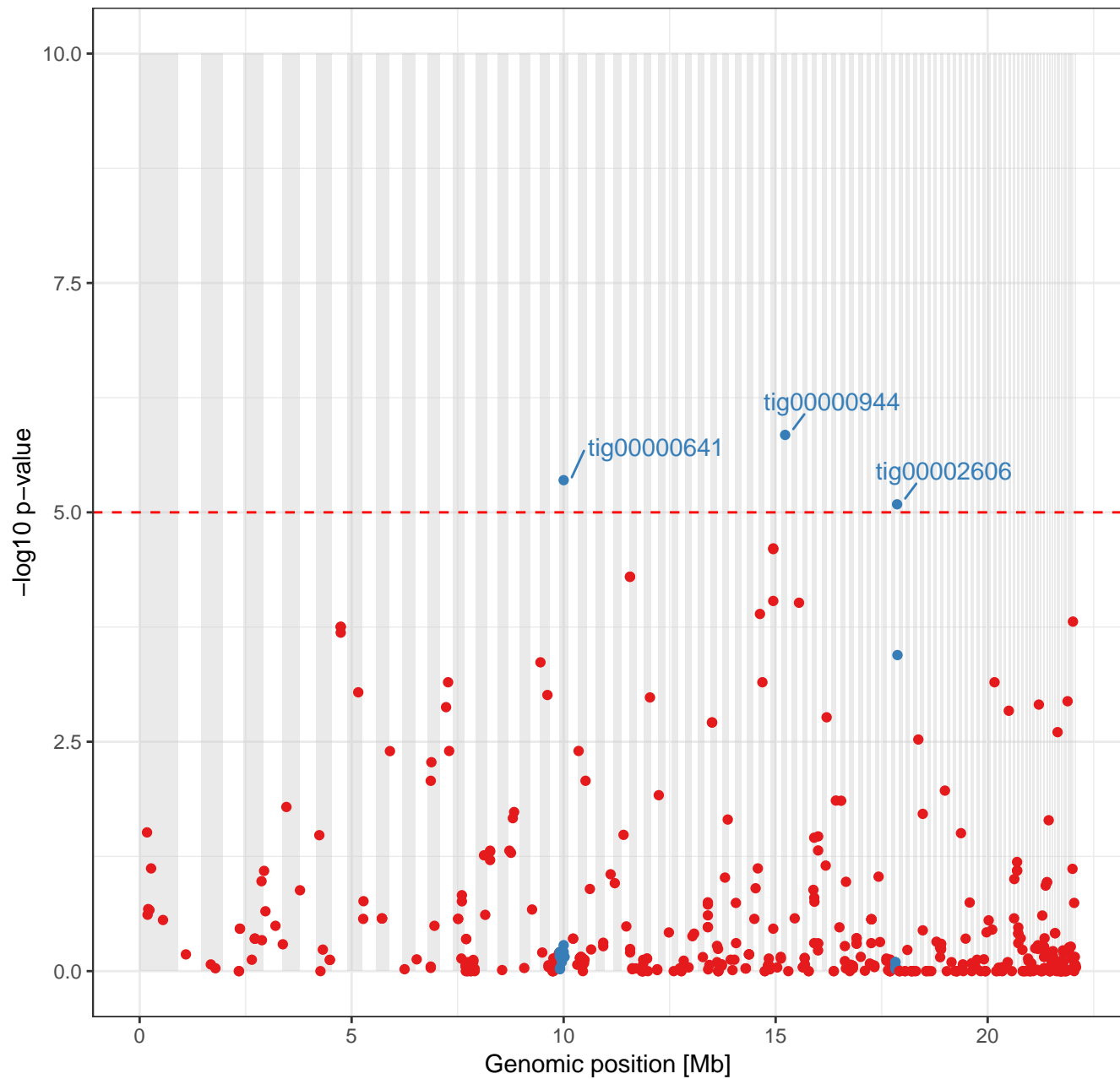

Supplement: evz219_Supplementary_Data [file evz219_supplementary_data.zip › fig_S6.pdf]

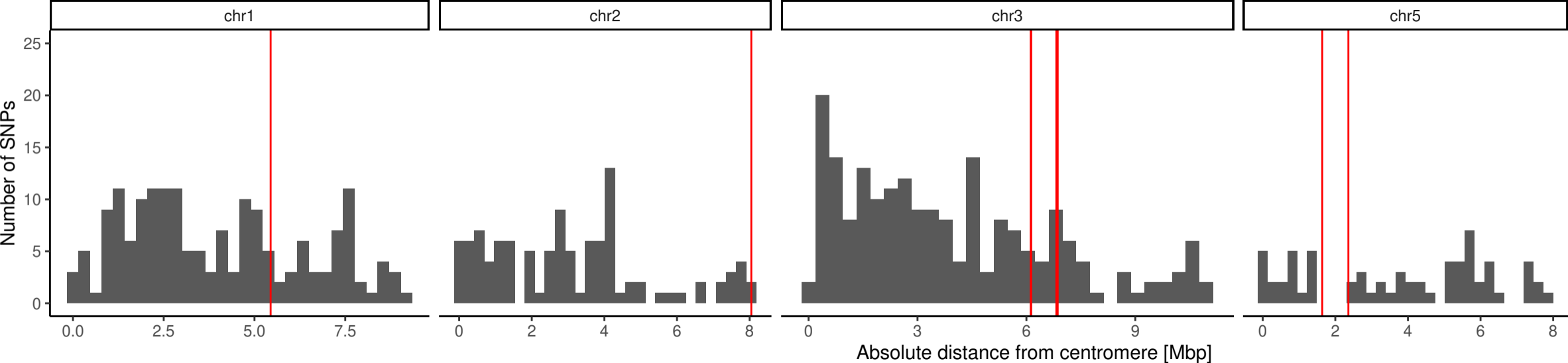

Supplement: evz219_Supplementary_Data [file evz219_supplementary_data.zip › fig_S7.pdf]
